# Advancements in Heart Failure Management: A Comprehensive Narrative Review of Emerging Therapies

**DOI:** 10.7759/cureus.46486

**Published:** 2023-10-04

**Authors:** FNU Sapna, FNU Raveena, Maria Chandio, Karoona Bai, Mohammad Sayyar, Giustino Varrassi, Mahima Khatri, Satesh Kumar, Tamam Mohamad

**Affiliations:** 1 Pathology, Albert Einstein College of Medicine, New York, USA; 2 Medicine, Ghulam Muhammad Mahar Medical College, Sukkur, PAK; 3 Medicine, Liaquat University of Medical and Health Sciences, Jamshoro, PAK; 4 Internal Medicine, Dow University of Health Sciences, Karachi, PAK; 5 Medicine, Khyber Medical College, Peshawar, PAK; 6 Pain Medicine, Paolo Procacci Foundation, Rome, ITA; 7 Medicine and Surgery, Dow University of Health Sciences, Karachi, PAK; 8 Medicine and Surgery, Shaheed Mohtarma Benazir Bhutto Medical College, Karachi, PAK; 9 Cardiovascular Medicine, Wayne State University, Detroit, USA

**Keywords:** cardiac care, comprehensive review, emerging therapies, advancements, management, heart failure

## Abstract

Heart failure is a substantial and escalating global health challenge, affecting millions worldwide. This complex syndrome arises from diverse etiologies, encompassing ischemic heart disease, hypertension, valvular abnormalities, and cardiomyopathies. Heart failure is characterized by the heart's inability to pump blood efficiently to meet the body's metabolic demands, leading to debilitating symptoms, frequent hospitalizations, and high mortality rates. Traditionally, the management of Heart failure has focused on alleviating symptoms, reducing fluid retention, and enhancing cardiac contractility. These goals have been achieved through a combination of pharmacological therapies such as angiotensin-converting enzyme inhibitors, beta-blockers, and diuretics, often complemented by device-based interventions like implantable cardioverter-defibrillators and cardiac resynchronization therapy. However, despite these advances, the relentless progression of heart failure remains a significant clinical challenge. Neurohormonal activation, cardiac fibrosis, and cellular remodeling are just a few of the intricate processes contributing to the disease's progression. In recent years, researchers and clinicians have embarked on a quest to identify novel therapeutic approaches that address these underlying mechanisms. One such avenue of exploration involves the revolutionary field of gene therapy, with promising gene-editing techniques, such as CRISPR-Cas9, offering potential routes for correcting genetic mutations that contribute to heart failure. Additionally, regenerative medicine approaches, including stem cell therapy and tissue engineering, hold significant promise for repairing damaged cardiac tissue and restoring function. Furthermore, precision medicine initiatives have gained traction, aiming to tailor heart failure therapies to individual patient profiles, taking into account genetics, biomarkers, and comorbidities. Integrating artificial intelligence and machine learning in heart failure management has also enabled the development of predictive models for early intervention, risk stratification, and personalized treatment recommendations. This narrative review navigates the intricate landscape of emerging therapies for heart failure, emphasizing their potential to revolutionize the field by targeting the disease's fundamental mechanisms. By exploring these innovative approaches, we aspire to provide a comprehensive perspective on the evolving paradigm of heart failure management, fostering a hopeful outlook for patients and clinicians alike.

## Introduction and background

Heart failure is a rapidly growing global health issue that significantly impacts individuals and healthcare systems around the globe. The disease in question is characterized by its intricate and diverse nature, resulting in a notable reduction in the overall well-being of those affected, as well as imposing a significant financial strain. This narrative review explores the crucial subject of "Advancements in Heart Failure Management," with a particular emphasis on developing therapies that have the potential to impact the field of heart failure care significantly. The significance of this narrative review resides not only in the critical assessment of emerging therapeutic strategies but also in the acknowledgment of heart failure as a significant public health concern necessitating innovative resolutions. To fully comprehend the importance of breakthroughs in heart failure management, it is imperative to grasp the scale and gravity of the issue. 

Heart failure significantly impacts many individuals globally, with around 64 million affected. Moreover, the prevalence of this condition is steadily increasing. The increase in occurrences of heart failure can be partially linked to the aging population and a higher incidence of cardiovascular risk factors such as hypertension, diabetes, and obesity [1]. Heart failure imposes a significant economic burden since healthcare costs related to this condition reach billions of dollars each year [2]. In addition to its financial implications, heart failure significantly diminishes the overall well-being of those afflicted. This is evidenced by the emergence of symptoms such as difficulty in breathing (dyspnea), persistent tiredness (fatigue), and reduced ability to engage in physical activities (exercise intolerance). Ultimately, these symptoms contribute to a notable decrease in life expectancy [3]. Heart failure therapy has evolved substantially in recent decades, influenced mainly by advancements in research and clinical practices. Pharmacological therapies, such as angiotensin-converting enzyme (ACE) inhibitors, beta-blockers, angiotensin receptor blockers (ARBs), and diuretics, have historically served as the fundamental approach to managing heart failure. The primary objective of these drugs is to mitigate symptoms, diminish fluid retention, and enhance heart function [4]. In addition, implantable devices such as implantable cardioverter-defibrillators (ICDs) and cardiac resynchronization therapy (CRT) have emerged as crucial instruments in the arsenal against heart failure, demonstrating significant reductions in mortality and hospitalizations among specific groups of patients [5,6]. Cardiac transplantation has consistently been recognized as the preferred treatment for people with end-stage heart failure, offering a vital opportunity for individuals with persistent symptoms and compromised cardiac performance [7]. Nevertheless, the limited availability of donor organs restricts its potential implementation.

Notwithstanding these noteworthy therapeutic advancements, heart failure continues to pose substantial obstacles. Notwithstanding the availability of current medicines, it is essential to acknowledge that not all patients exhibit ideal responses, and the disease frequently exhibits a persistent progression. The management of medical conditions can be further complicated by adverse effects, medication intolerances, and non-adherence to complex drug regimens [8]. A significant unmet need exists for alternative and complementary approaches to heart failure therapy. The continuous increase in the prevalence of heart failure and the constraints of existing treatments highlight the urgent requirement for developing novel therapeutic strategies. Emerging therapies comprise a diverse range of innovative methods, including advanced pharmacological agents, interventions based on devices, regenerative medicine efforts, and precision medicine projects. The principal objective of these nascent medicines is to enhance patient outcomes, optimize quality of life, and more efficiently target the underlying processes of heart failure. 

This narrative review thoroughly examines these emerging medicines, exploring their potential to transform heart failure management fundamentally. This review seeks to contribute to the existing knowledge on heart failure by comprehensively examining recent advancements and their potential consequences. The ultimate goal is to stimulate innovation and progress within the area. This study aims to thoroughly analyze the diverse developing medicines in managing heart failure, thoroughly examining their underlying mechanisms of action, clinical efficacy, and prospective benefits compared to established therapy modalities. Every developing therapy will undergo a rigorous evaluation, assessing its merits and inherent constraints. A comprehensive review of these therapeutic approaches will give physicians and researchers valuable insights to facilitate informed decision-making on their integration into clinical practice. The narrative review will finish by proposing prospective avenues for future study and advancement in heart failure management. Identifying topics necessitating further inquiry can serve as a valuable tool in guiding future research endeavors and promoting a culture of ongoing innovation. This narrative review aims to explore the diverse range of developing therapeutics for heart failure to provide a thorough examination of the breakthroughs that show potential in transforming the treatment of this widespread global health issue.

## Review

Methods

Search Strategy

A comprehensive search strategy was employed to ensure the comprehensiveness and rigor of this narrative review. Databases, including PubMed, Embase, Scopus, and Web of Science, were systematically searched for relevant articles, reviews, and clinical studies published up to this review's commencement (September 2023). Keywords such as "heart failure," "emerging therapies," "pharmacological advancements," "device-based interventions," "regenerative medicine," and "precision medicine" were utilized in various combinations to identify pertinent literature.

Inclusion and Exclusion Criteria

Inclusion criteria were established to select articles that align with the scope of this narrative review-including studies and articles needed to focus on emerging therapies in heart failure management, covering various aspects such as pharmacological innovations, device-based interventions, regenerative medicine, and precision medicine. Articles critically evaluating emerging therapies and their potential impact on clinical practice were prioritized. Exclusion criteria were applied to exclude articles that did not meet the specified scope, were not written in English, or were not peer-reviewed. Additionally, studies solely focused on basic science research without clinical relevance were excluded.

Data Synthesis

Data synthesis was performed by categorizing the emerging therapies into thematic groups, such as pharmacological advancements, device-based interventions, regenerative medicine, and precision medicine. Each category was analyzed individually, focusing on summarizing the mechanisms of action, clinical effectiveness, strengths, and limitations of the therapies.

Ethical Considerations

This narrative review adheres to ethical principles and guidelines for conducting systematic and narrative studies. No primary data collection from human subjects was performed for this review; therefore, ethical approval was not required. However, ethical considerations related to the use of patient data in the included studies were discussed where applicable.

Limitations

It is essential to acknowledge that narrative reviews, while valuable for synthesizing existing knowledge, are subject to limitations such as selection bias in article inclusion and potential bias in data interpretation. Efforts have been made to minimize these limitations through a systematic and transparent methodology.

Understanding heart failure

Heart failure is a multifaceted and debilitating clinical syndrome characterized by the heart's inability to pump blood effectively to meet the body's metabolic demands. It represents a significant global health challenge, impacting millions of individuals and straining healthcare systems worldwide. This comprehensive article aims to provide an extensive overview of heart failure, encompassing its definition, epidemiology, classification, and pathophysiology. It will also delve into the multifactorial nature of the disease and its challenges. Heart failure, also known as congestive heart failure, is a complex clinical syndrome marked by the heart's compromised ability to pump blood efficiently, resulting in inadequate circulation and oxygen delivery to meet the body's metabolic requirements. It is not a singular disease entity but rather a syndrome that arises from various cardiovascular and non-cardiovascular factors.

Epidemiology of Heart Failure

Heart failure is a pervasive health concern that affects millions of people worldwide. Its prevalence continues to rise, driven by aging populations, improved survival following heart attacks, and an increasing burden of risk factors, including hypertension, diabetes, and obesity. In the United States, it was estimated that approximately 6.2 million adults aged 20 and older had heart failure in 2019 [9]. Heart failure is associated with a substantial mortality and morbidity burden. Prognosis varies depending on the severity, underlying etiology, and access to healthcare. The one-year mortality rate following diagnosis can range from 20% to 30%, while the five-year mortality rate can exceed 50% for advanced heart failure [10].

Moreover, heart failure is a leading cause of hospitalization among older adults, resulting in a significant economic burden on healthcare systems [10]. The economic impact of heart failure is considerable. It places a substantial financial strain on healthcare systems due to recurrent hospitalizations, expensive interventions, and long-term management. Heart failure's estimated direct and indirect costs in the United States alone surpassed $30 billion in 2012 [11].

Classification of Heart Failure

Heart failure with reduced ejection fraction (HFrEF) vs. heart failure with preserved ejection fraction (HFpEF). HFrEF is defined by a left ventricular ejection fraction (LVEF) of less than 40%. It is often associated with impaired heart muscle contractile function and is more commonly attributed to conditions such as ischemic heart disease, cardiomyopathies, or myocarditis. HFpEF patients exhibit an LVEF more significantly than 50%. HFpEF is associated with impaired relaxation and increased left ventricle stiffness, often occurring in the context of hypertension, diabetes, or aging [11]. Acknowledging the limitations of the HFrEF and HFpEF classifications, the European Society of Cardiology introduced a third category known as heart failure with mid-range Ejection fraction (HFmrEF). This category encompasses patients with LVEF ranging from 40% to 49% and aims to address the heterogeneous nature of heart failure. This form occurs suddenly and often necessitates immediate medical attention. The rapid onset of symptoms typically characterizes it and may lead to acute decompensation, requiring hospitalization. Chronic heart failure is characterized by persistent and long-term symptoms [12]. It necessitates ongoing medical management to control symptoms and improve the patient's quality of life. 

Pathophysiology

Understanding the pathophysiology of heart failure is paramount for effective diagnosis and management. It involves a complex interplay of various factors, including structural, functional, and neurohormonal changes in the heart. In most cases of heart failure, there is a process known as left ventricular remodeling. This process involves alterations in the left ventricle's size, shape, and structure, often resulting from myocardial infarction, hypertension, or chronic volume overload. These structural changes can impair contractility and reduce stroke volume [13]. Chronic pressure overload, as seen in hypertension, induces ventricular hypertrophy. Initially, the increased thickness of the ventricular walls compensates for an increased workload. However, it can impair diastolic function over time and contribute to heart failure [14]. Systolic dysfunction, characterized by reduced ejection fraction, is a hallmark of HFrEF. It results from impaired myocardium contractility, which reduces the heart's ability to eject blood effectively during systole [14]. In HFpEF and, to some extent, HFmrEF, diastolic dysfunction is prominent. This involves impaired relaxation of the ventricles during diastole, leading to reduced ventricular filling and increased pressure. Diastolic dysfunction can result from myocardial fibrosis and increased ventricular stiffness [15].

Neurohormonal Activation

In response to reduced cardiac output, the renin-angiotensin-aldosterone system (RAAS) is activated. This leads to increased levels of angiotensin II and aldosterone, which cause vasoconstriction and sodium and water retention, ultimately increasing cardiac workload and exacerbating heart failure. Sympathetic nervous system (SNS) activation is another compensatory mechanism in heart failure. It leads to increased heart rate, contractility, and vasoconstriction, all of which are initially beneficial but can become detrimental in the long term [16]. In response to increased ventricular wall stretch, the heart releases natriuretic peptides (e.g., atrial natriuretic peptide or ANP and B-type natriuretic peptide or BNP). These peptides have diuretic and vasodilatory effects and act to counterbalance the adverse effects of the RAAS and SNS. Chronic inflammation and oxidative stress are often observed in heart failure. These processes can damage myocardial cells, lead to fibrosis, and further deteriorate cardiac function [17]. Endothelial dysfunction, characterized by impaired nitric oxide production and increased endothelin-1, is joint in heart failure. It contributes to vasoconstriction, increased afterload, and impaired coronary blood flow. At the cellular level, heart failure involves changes in calcium handling, alterations in myocyte structure, and shifts in gene expression patterns. These changes can lead to contractile dysfunction and apoptosis of myocardial cells [17].

Multifactorial Nature of Heart Failure

Heart failure is not a single disease entity; it is the final common pathway of various cardiac and non-cardiac conditions. This multifactorial nature makes both diagnosis and treatment complex. Coronary artery disease, myocardial infarction, and chronic ischemia significantly contribute to heart failure, particularly HFrEF. Dilated, hypertrophic, and restrictive cardiomyopathy can lead to heart failure. Conditions such as aortic stenosis and mitral regurgitation can overload the heart and eventually result in heart failure. Severe and poorly controlled arrhythmias can disrupt normal heart function and contribute to heart failure [18]. Chronic hypertension can lead to left ventricular hypertrophy and diastolic dysfunction, contributing to HFpEF. Uncontrolled diabetes is associated with an increased risk of heart failure, possibly due to microvascular and macrovascular changes. Obesity is a significant risk factor for heart failure, contributing to systolic and diastolic dysfunction. Chronic kidney disease and heart failure often coexist in a bidirectional relationship, with each condition exacerbating the other. Chronic obstructive pulmonary disease (COPD) and pulmonary hypertension can strain the right ventricle, leading to heart failure. Severe anemia can reduce oxygen-carrying capacity, placing additional strain on the heart. Excessive alcohol consumption, exposure to cardiotoxic medications, and illicit drug use can damage the myocardium and lead to heart failure [19]. There is also a genetic component to heart failure. Genetic mutations and familial predispositions can increase an individual's risk of heart failure, particularly in cardiomyopathies [18].

Current treatment landscape

The management of heart failure has evolved significantly over the past few decades, focusing on alleviating symptoms, improving quality of life, reducing hospitalizations, and prolonging survival. Treatment strategies are tailored to the type and stage of heart failure, with a primary division between HFrEF and HFpEF. This section will explore the existing treatments and management strategies for heart failure, including pharmacological interventions, device-based therapies, and lifestyle modifications. Additionally, it will highlight the limitations of current treatments and emphasize the ongoing need for innovation in heart failure management.

Pharmacological Interventions

ACE inhibitors have been a cornerstone in treating HFrEF. They reduced the production of angiotensin II, a potent vasoconstrictor, and aldosterone, leading to vasodilation and decreased sodium and water retention. Essential drugs in this class include enalapril, lisinopril, and ramipril. The landmark CONSENSUS trial demonstrated the significant mortality reduction of enalapril in patients with severe heart failure [20]. ARBs, such as losartan and valsartan, offer an alternative for patients intolerant to ACE inhibitors. They block the action of angiotensin II at the receptor level and have shown effectiveness in reducing morbidity and mortality in heart failure patients. Beta-blockers, including carvedilol, metoprolol, and bisoprolol, have emerged as a critical therapeutic option for HFrEF. These drugs antagonize the effects of catecholamines, reducing heart rate and myocardial oxygen consumption while improving contractility. The CIBIS-II trial demonstrated the efficacy of bisoprolol in reducing mortality in HFrEF patients. Mineralocorticoid receptor antagonists (MRAs), such as spironolactone and eplerenone, target aldosterone receptors, counteracting their sodium and water-retaining effects. The RALES trial highlighted the benefits of spironolactone in reducing mortality in severe HFrEF [21]. Sacubitril/valsartan, a combination of a neprilysin inhibitor (sacubitril) and an ARB (valsartan), represents a breakthrough therapy for HFrEF. It augments natriuretic peptide levels while blocking the detrimental effects of angiotensin II. The PARADIGM-HF trial demonstrated superior outcomes with sacubitril/valsartan compared to enalapril in reducing cardiovascular mortality and heart failure hospitalizations [21]. Diuretics, such as furosemide and hydrochlorothiazide, are essential for managing fluid overload in heart failure. They promote diuresis, alleviating symptoms of congestion. However, their long-term use may lead to electrolyte imbalances and renal dysfunction. Digoxin remains an option in selected heart failure cases, particularly for rate control in patients with atrial fibrillation. It has positive inotropic effects and can improve symptoms but does not significantly impact mortality. Ivabradine is a heart rate-lowering medication indicated for patients with HFrEF who remain symptomatic despite optimal medical therapy. It inhibits the funny current (If) in the sinoatrial node, reducing heart rate without adverse inotropic effects [21].

Device-Based Therapies

ICDs are recommended for select HFrEF patients at risk of sudden cardiac death, particularly those with a history of ventricular tachycardia or fibrillation. ICDs monitor the heart's rhythm and deliver shocks when life-threatening arrhythmias occur. CRT involves the implantation of a biventricular pacemaker to synchronize the contraction of the heart's ventricles. It benefits HFrEF patients with intraventricular conduction delays, improving symptoms and reducing mortality [22]. Left ventricular assist devices (LVADs) are mechanical pumps implanted in the chest to augment the heart's pumping function. They are used as a bridge to transplantation or as destination therapy in patients who are not transplant candidates [22].

Heart Transplantation

Heart transplantation is considered the ultimate therapy for end-stage heart failure when medical and device-based treatments are no longer effective. However, donor organ scarcity limits its availability.

Lifestyle Modifications and Non-Pharmacological Approaches

Dietary sodium restriction is a critical component of heart failure management. Limiting sodium intake helps reduce fluid retention and congestion, alleviating symptoms. Fluid restriction is often recommended to manage fluid overload in advanced heart failure. Patients are advised to monitor daily fluid intake carefully. For obese patients with heart failure, weight management and lifestyle changes are crucial. Achieving and maintaining a healthy body weight can improve heart function and reduce symptoms [18]. Regular exercise under the supervision of a healthcare provider is essential in managing heart failure. Exercise can improve cardiovascular fitness, muscle strength, and overall quality of life. Smoking cessation is paramount, as tobacco use exacerbates heart failure and increases the risk of cardiovascular events. For patients with alcohol-related heart failure, alcohol restriction or abstinence is essential to prevent further myocardial damage [19].

Limitations of Current Therapies

Many medications and device-based therapies have shown remarkable benefits in HFrEF but have limited efficacy in HFpEF, for which there is currently no disease-modifying therapy. Drugs such as ACE inhibitors and beta-blockers can cause side effects, including hypotension, renal dysfunction, and hyperkalemia, necessitating careful monitoring. Some patients may develop tolerance or resistance to medications over time, necessitating dosage adjustments or the addition of alternative therapies. Device-based therapies, such as LVADs and ICDs, carry risks of complications, including infections, bleeding, and device malfunction. Access to advanced treatments such as heart transplantation and LVADs is limited by donor organ availability and cost considerations [23]. Successful heart failure management relies heavily on patient adherence to complex medication regimens, lifestyle modifications, and follow-up appointments, which can be challenging.

Ongoing Need for Innovation

Advancements in precision medicine may lead to tailored therapies based on an individual's genetic, molecular, and clinical profile. This approach can optimize treatment efficacy while minimizing side effects. The search for novel pharmacological agents continues, with ongoing research into drugs targeting specific molecular pathways involved in heart failure pathophysiology [24]. Biomarker-based approaches aim to identify heart failure earlier, allowing for timely intervention and risk stratification. Promising biomarkers include galectin-3, soluble ST2, and high-sensitivity troponins [25]. Telemedicine and remote monitoring technologies can improve heart failure management by facilitating real-time patient data collection, tracking medication adherence, and early intervention for symptom exacerbation. Regenerative therapies, such as stem cell and gene-based therapies, hold promise for repairing damaged myocardium and restoring cardiac function. Advanced mechanical support devices aim to improve the durability, portability, and safety of devices like LVADs, making them accessible to a broader range of patients. Artificial intelligence and machine learning algorithms are being developed to predict heart failure exacerbations, optimize medication regimens, and assist in personalized treatment plans [25]. Heart failure is a complex and multifactorial syndrome with a significant global burden. It necessitates a comprehensive understanding of its epidemiology, classification, and pathophysiology for effective management. Current treatments, including pharmacological interventions, device-based therapies, and lifestyle modifications, have improved outcomes for many heart failure patients. However, they have limitations, and there is a pressing need for ongoing innovation to address the diverse and evolving challenges posed by this condition. As research advances and new therapeutic avenues are explored, the hope is that heart failure management will become increasingly personalized, effective, and accessible, ultimately improving the quality of life and prognosis for individuals affected by this debilitating syndrome.

Pharmacological innovations

Heart failure is a complex and prevalent cardiovascular syndrome with a substantial global burden. Despite significant progress in treatment options, managing heart failure remains challenging due to its multifactorial etiology and diverse clinical manifestations. In recent years, pharmacological innovations have played a crucial role in improving the prognosis and quality of life for patients with heart failure. This section will discuss the latest advancements in pharmacological therapies, focusing on novel drug classes, mechanisms of action, and clinical trial findings.

Novel Drug Classes

Sodium-glucose co-transporter 2 (SGLT2) inhibitors, originally developed for treating diabetes mellitus, have emerged as a groundbreaking class of drugs in heart failure management. These medications, including empagliflozin, dapagliflozin, and canagliflozin, work by inhibiting glucose reabsorption in the renal proximal tubules, leading to glycosuria and improved glycemic control in diabetic patients. However, their benefits in heart failure go beyond glycemic control. SGLT2 inhibitors exert several beneficial effects on the cardiovascular system. They promote diuresis and natriuresis, reducing blood volume and cardiac preload. Additionally, they improve myocardial energetics by increasing the utilization of ketone bodies as an alternative fuel source for the heart. Furthermore, SGLT2 inhibitors reduce oxidative stress and inflammation while improving endothelial function [25]. The EMPA-REG OUTCOME trial, involving empagliflozin, demonstrated a significant reduction in cardiovascular death, hospitalization for heart failure, and all-cause mortality in patients with type 2 diabetes and established cardiovascular disease. Subsequently, the EMPEROR-Reduced and EMPEROR-Preserved trials evaluated empagliflozin in heart failure patients with reduced ejection fraction (HFrEF) and preserved ejection fraction (HFpEF), respectively. Both trials showed reduced heart failure hospitalizations and improved renal outcomes [24]. Dapagliflozin studied in the DAPA-HF trial, also reduced heart failure-related events in HFrEF patients, irrespective of their diabetic status [24]. These findings have led to the inclusion of SGLT2 inhibitors in the guidelines for managing HFrEF. Angiotensin receptor-neprilysin inhibitors (ARNIs) represent another innovative class of drugs for heart failure management. Sacubitril/valsartan, an ARNI, combines a neprilysin inhibitor with an angiotensin receptor blocker (ARB). Neprilysin is an enzyme responsible for degrading natriuretic peptides, which have vasodilatory and diuretic effects. By inhibiting neprilysin, sacubitril/valsartan increases the levels of natriuretic peptides, leading to vasodilation, reduced ventricular remodeling, and improved diuresis. The concurrent blockade of the RAAS by valsartan provides additional benefits [22]. The PARADIGM-HF trial compared sacubitril/valsartan with enalapril in HFrEF patients and demonstrated a significant reduction in cardiovascular mortality, heart failure hospitalizations, and all-cause mortality with sacubitril/valsartan [21]. This landmark trial led to the approval of sacubitril/valsartan as a first-line therapy for HFrEF. Vericiguat, a soluble guanylate cyclase (sGC) stimulator, is a recent addition to the armamentarium of heart failure medications. sGC stimulators enhance the activity of sGC, a key enzyme involved in the nitric oxide (NO) signaling pathway. By increasing cyclic guanosine monophosphate (cGMP) production, vericiguat promotes vasodilation, reduces myocardial hypertrophy, and improves cardiac relaxation and compliance. This class of drugs is up-and-coming in HFpEF, where impaired NO signaling plays a pivotal role [23]. The VICTORIA trial assessed the efficacy of vericiguat in reducing cardiovascular death and heart failure hospitalization in HFrEF patients. It showed a significant reduction in these endpoints, supporting the use of vericiguat in this patient population [23]. Vericiguat represents a novel approach to managing HFrEF by targeting the NO-cGMP pathway. Understanding the mechanisms underlying these pharmacological innovations is crucial for optimizing their use in heart failure management. The following section delves into the mechanistic insights that drive these novel drug classes. While initially developed for diabetes management, SGLT2 inhibitors have demonstrated remarkable cardiovascular benefits in heart failure patients. Their mechanism of action extends beyond glycemic control to target several key pathophysiological processes in heart failure. SGLT2 inhibitors promote diuresis by inhibiting glucose and sodium reabsorption in the renal tubules. This effect reduces blood volume and cardiac preload, alleviating congestion and symptoms of heart failure. Heart failure is characterized by impaired myocardial energetics, with a shift towards increased reliance on glucose metabolism. SGLT2 inhibitors encourage the use of ketone bodies as an energy source for the heart, potentially improving cardiac efficiency and function [24]. SGLT2 inhibitors have been shown to reduce oxidative stress and inflammation, which are implicated in the progression of heart failure. These drugs improve endothelial function, enhancing nitric oxide (NO) bioavailability and vasodilation. SGLT2 inhibitors reduce intraglomerular pressure and mitigate the risk of acute kidney injury, a common complication in heart failure patients [25]. Sacubitril/valsartan, an ARNI, combines neprilysin inhibition with angiotensin receptor blockade. The dual mechanism of action offers synergistic benefits in heart failure management. Neprilysin is responsible for the degradation of natriuretic peptides, which have vasodilatory, diuretic, and antiproliferative effects. Inhibiting neprilysin increases the levels of these peptides, promoting vasodilation, reducing ventricular remodeling, and enhancing diuresis [25]. Valsartan, an angiotensin receptor blocker, simultaneously inhibits the RAAS. RAAS activation contributes to vasoconstriction, sodium and water retention, and ventricular remodeling in heart failure. By blocking the RAAS, valsartan helps counter these deleterious effects [25]. Vericiguat, a guanylate cyclase modulator, targets the nitric oxide (NO)-cyclic guanosine monophosphate (cGMP) pathway, which plays a pivotal role in cardiovascular regulation. In heart failure, NO signaling is impaired, contributing to vasoconstriction, ventricular hypertrophy, and diastolic dysfunction. Vericiguat stimulates soluble guanylate cyclase (sGC), enhancing the production of cGMP and restoring NO signaling, which leads to vasodilation, reduced hypertrophy, and improved diastolic function [24]. Increased cGMP levels result in vasodilation, reducing afterload and myocardial workload, which is particularly beneficial in HFrEF.

Clinical Implications and Guidelines

The emergence of these novel drug classes has profoundly impacted heart failure management and prompted updates to clinical guidelines. Here, we explore these innovative pharmacological therapies' clinical implications and guideline recommendations. The remarkable benefits of SGLT2 inhibitors in heart failure have led to their inclusion in clinical guidelines. The American College of Cardiology (ACC) and American Heart Association (AHA) 2021 guidelines for managing heart failure recommend using SGLT2 inhibitors in patients with HFrEF, irrespective of their diabetic status. Specifically, dapagliflozin and empagliflozin are recommended to reduce the risk of heart failure, hospitalization, and cardiovascular death [17]. These guidelines reflect the transformative potential of SGLT2 inhibitors in improving outcomes for heart failure patients. Healthcare providers are increasingly incorporating these medications into their treatment regimens, especially for patients with HFrEF. Sacubitril/valsartan, the prototypical ARNI, has received widespread recognition as a cornerstone therapy for HFrEF. The AHA/ACC guidelines recommend sacubitril/valsartan as a replacement for ACE inhibitors or ARBs in patients with chronic HFrEF who remain symptomatic despite optimal treatment with an ACE inhibitor, ARB, or ARNI [17]. These guidelines emphasize the importance of transitioning eligible HFrEF patients to sacubitril/valsartan for better outcomes and symptom relief. The therapy's dual mechanism of action, targeting neprilysin and the RAAS, offers a unique advantage in reducing morbidity and mortality.

Challenges and Considerations

While these novel pharmacological innovations offer promising therapeutic options for heart failure patients, several challenges and considerations must be addressed. The cost of these innovative medications may pose a barrier to access for some patients, particularly in healthcare systems with limited resources. Ensuring affordability and equitable access to these therapies is essential to maximize their impact on heart failure outcomes. Optimal patient selection is crucial to realizing the benefits of these drugs while minimizing potential risks [18]. Clinicians must consider individual patient characteristics, including comorbidities, medication interactions, and contraindications when prescribing these therapies [18]. The long-term safety profile of these novel drugs requires ongoing evaluation, particularly regarding potential adverse effects and interactions with other medications. Post-marketing surveillance and real-world studies will provide valuable insights into their safety. Healthcare providers and patients need comprehensive education and awareness regarding these drugs' mechanisms, benefits, and potential side effects. This knowledge empowers shared decision-making and adherence to treatment plans [19].

Device-based interventions in heart failure management

In addition to pharmacological innovations, device-based interventions have been pivotal in advancing heart failure management. These interventions encompass a wide range of technologies, including implantable devices, remote monitoring systems, and artificial intelligence (AI) applications. This section explores the role of innovative devices in heart failure management and their impact on patient outcomes.

Implantable Devices

CRT involves the implantation of a specialized pacemaker with the capacity to simultaneously pace both the left and right ventricles. CRT is primarily indicated for heart failure patients with dyssynchrony, characterized by delayed or uncoordinated contraction of the ventricles. CRT aims to synchronize ventricular contraction, which can significantly improve cardiac function in patients with left bundle branch block or intraventricular conduction delays. By optimizing the timing of ventricular contractions, CRT enhances stroke volume, reduces mitral regurgitation, and increases cardiac output [20]. CRT has been shown to improve symptoms, exercise capacity, and quality of life in patients with heart failure, particularly those with HFrEF. Additionally, CRT reduces hospitalizations and mortality in eligible candidates [21]. ICDs are electronic devices implanted under the skin that continuously monitor the heart's rhythm. They are designed to detect and terminate life-threatening ventricular arrhythmias, such as ventricular tachycardia and ventricular fibrillation. ICDs deliver high-energy electrical shocks to the heart when they see dangerous arrhythmias, restoring normal rhythm and preventing sudden cardiac death. They also have pacing capabilities for bradycardia and can store data on arrhythmia events for later analysis. ICDs have significantly reduced the risk of sudden cardiac death in patients at high risk for ventricular arrhythmias, such as those with prior myocardial infarction or symptomatic heart failure [22]. They are a vital component of the management of HFrEF.

Ventricular Assist Devices (VADs)

Ventricular assist devices (VADs) are mechanical pumps implanted in patients with advanced heart failure as a bridge to transplant or destination therapy. They can assist the weakened heart by pumping blood from the left ventricle to the aorta, augmenting cardiac output. VADs are designed to provide circulatory support, reducing the workload on the failing heart. Depending on the type of VAD, they can support the left ventricle, right ventricle, or both. VADs are used in patients with end-stage heart failure when other treatments have failed [23]. VADs can significantly improve survival and quality of life in patients with advanced heart failure who are not candidates for heart transplantation [24]. They have revolutionized the management of end-stage heart failure and offer hope to previously considered untreatable patients.

Remote Monitoring

Remote patient monitoring (RPM) involves using technology to track a patient's health status and transmit data to healthcare providers without frequent in-person visits. RPM is particularly relevant in heart failure management, enabling proactive monitoring of vital signs, symptoms, and medication adherence. RPM systems typically consist of wearable devices, such as wireless scales, blood pressure cuffs, and smartphones, that collect metrics like weight, blood pressure, heart rate, and oxygen saturation. This data is transmitted securely to healthcare providers, allowing for early detection of patient condition changes [25]. RPM has been associated with reduced hospitalizations and improved outcomes in heart failure patients. Timely intervention based on remote data can prevent exacerbations and optimize medication adjustments.

Implantable Hemodynamic Monitors

Implantable hemodynamic monitors are small devices implanted in the pulmonary artery. They continuously measure pulmonary artery pressures, providing real-time data on a patient's hemodynamic status. This information can guide treatment decisions and help prevent heart failure exacerbations. Implantable hemodynamic monitors use a wireless sensor to measure pressures in the pulmonary artery. Changes in these pressures can indicate worsening heart failure. Data from the sensor are transmitted to a remote monitoring system accessible by healthcare providers [21]. Studies have shown that the use of implantable hemodynamic monitors is associated with a reduction in heart failure hospitalizations. These devices provide valuable insights into a patient's condition, allowing for timely interventions and adjustments to treatment plans [17].

Artificial Intelligence Applications

Machine learning (ML) and AI have gained traction in heart failure management for risk prediction. ML algorithms can analyze vast datasets, including clinical, imaging, and laboratory data, to identify patterns and predict which patients are at higher risk of heart failure exacerbations. ML models are trained on large datasets to recognize subtle ways and associations that may not be apparent to human clinicians. These models can integrate various data sources, such as electronic health records, wearable device data, and genetic information, to generate risk scores and identify patients who may benefit from more intensive monitoring or interventions [15]. ML-based risk prediction models have the potential to enhance personalized medicine by identifying patients at higher risk of adverse events. This allows healthcare providers to allocate resources more efficiently and tailor interventions to individual patient needs. Natural language processing (NLP) is a branch of AI that focuses on understanding and processing human language. In heart failure management, NLP can be used to analyze unstructured clinical notes and medical literature to extract valuable insights. NLP algorithms can parse clinical narratives, extracting information on symptoms, medication adherence, and patient-reported outcomes. By analyzing these narratives, NLP can provide a more comprehensive understanding of a patient's condition and response to treatment [16]. NLP can enhance the depth and quality of clinical assessments by extracting valuable information from free-text clinical notes. This information can inform treatment decisions and facilitate more personalized care.

Clinical Implications and Guidelines

Device-based interventions and AI applications have introduced transformative approaches to heart failure management. CRT, ICDs, and VADs have well-established roles in heart failure management. Clinical guidelines, such as those from the American College of Cardiology, American Heart Association, and European Society of Cardiology, provide recommendations on patient selection and appropriate use of these devices. These guidelines emphasize the importance of multidisciplinary heart failure teams to evaluate patients for device therapy and ensure optimal outcomes [17]. Remote monitoring, including RPM and implantable hemodynamic monitors, is gaining recognition in heart failure management. While guidelines acknowledge the potential benefits of these technologies, they also emphasize the need for structured care programs and effective data integration into clinical workflows. This approach ensures that remote monitoring data are acted upon promptly to prevent heart failure exacerbations. AI applications like ML for risk prediction and NLP for text analysis are still emerging in heart failure management. Clinical guidelines still need to incorporate specific recommendations for AI use. However, as AI technologies mature and demonstrate their clinical utility, policies will likely evolve to guide their integration into practice [17]. The widespread adoption of implantable devices and remote monitoring systems can be limited by issues related to patient access, especially in underserved populations. Addressing disparities in access to these technologies is essential to ensure equitable care for all heart failure patients. Health data collection and transmission, particularly in remote monitoring and AI applications, raise concerns about data security and patient privacy. Healthcare systems must implement robust data protection measures to safeguard patient information. Integrating device-based interventions and AI applications into clinical practice requires healthcare workflow and practice changes [18]. This may involve training healthcare providers, developing standardized protocols, and establishing clear pathways for acting on remote monitoring data. While these technologies hold promise, their integration into clinical practice should be evidence-based. Continuous evaluation of their impact on patient outcomes is essential to ensure they provide tangible benefits regarding reduced hospitalizations, improved quality of life, and extended survival [19]. 

Regenerative medicine and stem cell therapy in heart failure

Heart failure is a complex and debilitating condition characterized by the inability of the heart to pump blood effectively, leading to symptoms such as fatigue, shortness of breath, and fluid retention. It is a primary global health concern with limited treatment options, particularly for patients with advanced disease [18]. In recent years, regenerative medicine and stem cell therapy have emerged as promising approaches to repair damaged cardiac tissue and improve heart function. This section explores the potential of regenerative medicine and stem cell therapy in the context of heart failure.

Stem Cells and Cardiac Regeneration

Stem cells are undifferentiated cells with the unique ability to differentiate into various cell types and promote tissue repair and regeneration. Embryonic stem cells (ESCs) are pluripotent stem cells derived from early-stage embryos. They can differentiate into all cell types, including cardiomyocytes, the specialized muscle cells of the heart. When introduced into the damaged heart, ESCs can develop into new cardiomyocytes, replacing the damaged tissue and improving cardiac function. Additionally, they may exert paracrine effects by releasing growth factors and cytokines that promote tissue repair [16]. The use of ESCs has been limited due to ethical concerns related to their derivation from embryos and the risk of teratoma formation. These challenges have led to the exploration of alternative stem cell sources. Induced pluripotent stem cells are generated by reprogramming adult somatic cells, such as skin cells, into a pluripotent state. iPSCs share similar differentiation capabilities with ESCs but can be derived from a patient's cells, reducing the risk of immune rejection. iPSCs can be differentiated into cardiomyocytes and other cardiac cell types, offering a potential source of patient-specific cells for cardiac regeneration. This approach holds promise for personalized regenerative therapies [7]. Induced pluripotent stem cell (iPSC)-based treatments face challenges related to safety, scalability, and the potential for genomic instability during reprogramming. Researchers continue to work on optimizing iPSC generation and differentiation protocols. Mesenchymal stem cells are multipotent stem cells found in various tissues, including bone marrow, adipose tissue, and umbilical cord blood. They have been extensively studied for their regenerative properties in heart failure. MSCs exhibit paracrine effects by secreting factors that modulate inflammation, promote angiogenesis (forming new blood vessels), and stimulate endogenous cardiac repair mechanisms. They can also differentiate into various cell types, including cardiomyocytes [8]. Numerous clinical trials have explored the safety and efficacy of MSC-based therapies in heart failure patients. While results regarding safety and some functional improvements have been promising, the mechanisms of action and long-term outcomes are still under investigation [9]. Cardiac progenitor cells are a specialized subset of stem cells within the heart. They are believed to play a role in cardiac development and repair. CPCs have the potential to differentiate into cardiomyocytes and contribute to cardiac regeneration. They may also secrete factors that promote tissue repair and modulate the local microenvironment. Early-phase clinical trials have explored the use of CPCs in heart failure patients, with some evidence of functional improvements. However, further research is needed to optimize CPC isolation and delivery methods [10].

Current Status of Stem Cell Therapy in Heart Failure

While stem cell therapy holds great promise for heart failure treatment, it is essential to acknowledge the current status of these therapies in clinical practice. Many clinical trials have demonstrated the safety and feasibility of stem cell-based interventions in heart failure. These studies have shown that stem cell transplantation, including autologous (patient's own) and allogeneic (donor-derived) cells, is generally well-tolerated and associated with low rates of adverse events. Some clinical trials have reported functional improvements in heart failure patients treated with stem cells. These improvements include increased LVEF, reduced ventricular remodelling, and enhanced exercise capacity [11]. The outcomes of stem cell therapy in heart failure have varied among patients and studies. Factors such as cell type, delivery method, timing of intervention, and patient selection criteria may influence treatment responses [12]. While short-term improvements have been observed in some trials, long-term efficacy and durability of stem cell-based interventions remain areas of ongoing investigation. Understanding the mechanisms of cell engraftment, survival, and functional integration is critical [12]. The optimal stem cell type and source for heart regeneration have yet to be definitively identified. Comparative studies are needed to determine which cell types offer the most robust regenerative potential. Allogeneic stem cell transplantation may elicit immune responses in recipients, potentially limiting the long-term success of therapy. Strategies to mitigate immune rejection are under investigation. Stem cell-based therapies involve complex regulatory and ethical considerations, mainly when using cells derived from embryos or manipulating the patient's cells. Ensuring patient safety and ethical use of stem cells are paramount.

Future Directions in Stem Cell Therapy

Despite the challenges, stem cell therapy remains promising for heart failure treatment. Ongoing research efforts aim to address the limitations and optimize the therapeutic potential of stem cells. Future directions in stem cell therapy for heart failure include. Advances in iPSC technology enable the generation of patient-specific cardiomyocytes. These cells can be used for disease modeling, drug testing, and potentially autologous transplantation, offering a personalized approach to heart regeneration [13]. Combining stem cells with biomaterials and tissue engineering techniques holds promise for creating functional cardiac patches or constructs. These engineered tissues could repair damaged myocardium and improve cardiac function [11]. Emerging research explores the potential synergies of combining stem cell therapy with other approaches, such as gene therapy, growth factors, or pharmacological agents. These combination therapies aim to enhance the regenerative effects and improve outcomes [13].

Clinical implementation and challenges

Implementing emerging therapies in clinical practice presents challenges and hurdles across various domains, including cost, accessibility, and ethical considerations. These challenges are pivotal factors influencing the successful integration of innovative medical treatments into the healthcare ecosystem. This discussion will explore these challenges in-depth, emphasizing their significance and impact. One of the most prominent challenges of implementing emerging therapies is their substantial financial burden. Developing novel therapies is a lengthy and expensive process. Pharmaceutical companies invest significant resources in research, preclinical studies, and rigorous clinical trials to bring innovative treatments to the market. These development costs are eventually reflected in the price of the therapies [15]. Emerging therapies, such as gene and personalized medications, often require complex manufacturing processes. This complexity drives production costs and can pose logistical challenges [14]. Even when emerging therapies gain regulatory approval, healthcare systems or insurers cannot guarantee immediate reimbursement. Evaluating cost-effectiveness and clinical efficacy can be lengthy, delaying patient access to these therapies. High treatment costs can translate into substantial out-of-pocket expenses for patients. Copayments, deductibles, and other financial obligations can significantly burden individuals and their families. The cost of emerging therapies can exacerbate existing healthcare disparities. Vulnerable populations with limited access to healthcare resources, such as lower-income individuals, may face substantial barriers to accessing these treatments, thereby perpetuating health inequalities [15].

Access Challenges

Ensuring equitable access to emerging therapies represents a significant challenge in healthcare. Geographic location can significantly influence access to emerging therapies. Patients in urban areas with well-funded healthcare systems may have more extensive access to cutting-edge treatments than those in rural or underserved regions, where healthcare infrastructure may be less robust [22]. Access to emerging therapies often depends on the availability of comprehensive insurance coverage. Patients with extensive insurance plans may find it easier to access these treatments, while those with limited or no insurance may face significant challenges [23]. Clinical trials are a critical pathway for patients to access emerging therapies. However, disparities exist in clinical trial participation, with underrepresented populations, such as racial and ethnic minorities, often having limited opportunities to participate [25]. Even after regulatory approval, the complexities of navigating regulatory pathways and securing reimbursement approvals can limit patient access to emerging therapies, especially for individuals with life-threatening or rapidly progressing conditions [16]. The delivery of emerging therapies often necessitates specialized expertise. Not all healthcare facilities and providers possess the knowledge and infrastructure to offer these treatments, requiring patients to travel considerable distances to access centers of excellence [18].

Ethical Considerations

The ethical dimensions of implementing emerging therapies in clinical practice are multifaceted and require careful consideration. Obtaining informed consent is a fundamental moral requirement in healthcare. Patients must receive clear, understandable information about emerging therapies, including potential risks, benefits, and alternatives. The novelty and complexity of these treatments can pose challenges in achieving fully informed consent. Many emerging therapies involve collecting and analyzing sensitive patient data, including genetic information and personal health records. Ensuring the privacy and security of this data is paramount, and healthcare institutions and researchers must implement robust data protection measures [16]. The allocation of limited healthcare resources, including emerging therapies, raises ethical questions about fairness and equity. Decisions regarding who receives access to these treatments can be ethically complex, particularly when demand surpasses supply [17]. The ethical principle of non-maleficence emphasizes the importance of avoiding harm to patients. Emerging therapies, while promising, may carry unknown risks or unanticipated side effects. Ethical considerations require ongoing monitoring and transparency regarding potential damages. Equity and justice are core ethical principles in healthcare. The adoption of emerging therapies must prioritize equitable access, regardless of factors such as race, socioeconomic status, or geographic location. Ethical frameworks should guide decision-making to minimize disparities [18]. These ethical considerations are essential in guiding healthcare providers, researchers, and policymakers to ensure that emerging therapies are implemented responsibly and ethically, focusing on patient well-being and equitable access.

Clinical implications and future directions

Emerging therapies in managing heart failure hold substantial promise, with the potential to profoundly impact patient care, clinical decision-making, and the broader landscape of heart failure management. This discussion delves into these therapies' clinical implications and explores future research and development directions.

Clinical Implications

Integrating emerging therapies into the clinical management of heart failure carries several significant clinical implications. Emerging therapies enable the tailoring of treatment plans to individual patient profiles. Healthcare providers can utilize genetic information, biomarkers, and clinical data to select the most appropriate therapies and monitor their effectiveness. This personalized approach can improve outcomes and reduce the risk of adverse events. Precision medicine facilitated by emerging therapies allows for more accurate risk prediction and early intervention. By identifying patients at higher risk of heart failure exacerbations or adverse outcomes, healthcare providers can proactively adjust treatment strategies and lifestyle recommendations to prevent complications. Emerging therapies, including novel drugs and stem cell-based treatments, offer the possibility of targeted interventions that address the underlying mechanisms of heart failure. As our understanding of the disease's molecular basis deepens, we can expect the development of more precise and effective treatments [19]. Data-driven clinical decision-making, supported by artificial intelligence and machine learning, is becoming increasingly important. These technologies can analyze vast datasets to identify patterns, predict outcomes, and assist healthcare providers in making informed choices about patient care [10]. Integrating emerging therapies and precision medicine necessitates a multidisciplinary approach to patient care. Heart failure teams may include cardiologists, genetic counselors, pharmacists, and other specialists collaborating to deliver comprehensive, individualized care. Patient engagement and empowerment are central to the success of emerging therapies and precision medicine. Informed patients who actively participate in their care can make decisions that align with their values and preferences, ultimately leading to better outcomes [15]. The field of heart failure management is dynamic and continually evolving. Research and development efforts will likely refine existing therapies, identify new targets, and explore innovative approaches to improve patient care. Collaboration between researchers, clinicians, and industry partners will drive innovation.

Future Directions

As emerging therapies continue to evolve, several future directions and areas of research and development can be anticipated. AI and machine learning algorithms can analyze vast datasets to identify complex patterns and predict patient outcomes [12]. These technologies hold promise for refining risk prediction models and guiding treatment decisions. Empowering patients to participate in their care actively is a central tenet of precision medicine. Patient-reported outcomes and preferences can inform treatment decisions and enhance shared decision-making. Integrating genomics, proteomics, metabolomics, and other omics data into clinical practice will provide a more comprehensive view of an individual's health. These data sources can uncover novel biomarkers and therapeutic targets [5]. Advancements in understanding the molecular mechanisms of heart failure may lead to the development of targeted therapies that address specific disease subtypes. These therapies can be tailored to individual patient profiles. While clinical trials provide valuable insights, real-world evidence is needed to assess emerging therapies' long-term safety and effectiveness. Post-marketing surveillance, patient registries, and comparative effectiveness research contribute to understanding how these therapies perform in real-world clinical settings [6]. As the adoption of emerging therapies increases, conducting cost-effectiveness studies will be crucial. Evaluating the economic impact of these therapies in real-world healthcare systems will inform resource allocation and reimbursement decisions. Future research may explore the benefits of combining emerging therapies with established treatments [7]. Combinations of pharmacological innovations, device-based interventions, and regenerative medicine could offer synergistic effects in managing heart failure. Ongoing research efforts will likely focus on discovering novel biomarkers associated with heart failure [10]. These biomarkers could improve diagnosis, risk stratification, and treatment guidance. Advances in gene editing technologies like CRISPR-Cas9 may open up new possibilities for modifying the genetic underpinnings of heart failure. Research in this area could lead to innovative gene therapies. The integration of emerging therapies may necessitate changes in healthcare delivery models. Research on optimizing care pathways, telehealth applications, and patient navigation systems can enhance the delivery of precision medicine.

## Conclusions

In conclusion, this review has highlighted the multifaceted nature of heart failure management, exploring its complexities, challenges, and promising innovations. The key takeaways underscore the transformative potential of ongoing advancements in heart failure management. These developments offer hope for patients facing this challenging condition, from novel pharmacological interventions to innovative device-based therapies, regenerative medicine, and precision care. These advancements enable tailored treatment plans, precise risk prediction, and a shift towards more patient-centric care. The fusion of data-driven clinical decision-making, multidisciplinary collaboration, and patient empowerment promises improved outcomes and enhanced quality of life for individuals with heart failure. As we navigate the challenges of cost, accessibility, and ethical considerations, it is clear that the pursuit of emerging therapies and precision medicine in heart failure management is not only a scientific endeavor but also a moral imperative. The future of heart failure care holds the potential to redefine the patient experience, reduce disparities, and, ultimately, save lives. With ongoing research and dedication, we can look forward to a brighter horizon for heart failure patients and a revolutionized care landscape.
